# The Effect of Additional Training on Motor Outcomes at Discharge from Recovery Phase Rehabilitation Wards: A Survey from Multi-Center Stroke Data Bank in Japan

**DOI:** 10.1371/journal.pone.0091738

**Published:** 2014-03-13

**Authors:** Nariaki Shiraishi, Yusuke Suzuki, Daisuke Matsumoto, Seungwon Jeong, Motoya Sugiyama, Katsunori Kondo, Masafumi Kuzuya

**Affiliations:** 1 Department of Geriatrics, Medicine in Growth and Aging, Program in Health and Community Medicine, Nagoya University Graduate School of Medicine, Nagoya, Japan; 2 Department of Rehabilitation, Faculty of Health Science, Nihon Fukushi University, Nagoya, Japan; 3 Department of Comprehensive Community Care Systems, Nagoya University Graduate School of Medicine, Nagoya, Japan; 4 Department of Physical Therapy, Faculty of Health Science, Kio University, Koryo, Japan; 5 Department of Social Science Center for Gerontology and Social Science, Chubu Rosai Hospital, Nagoya, Japan; 6 Department of Rehabilitation, Chubu Rosai Hospital, Nagoya, Japan; 7 Center for Well-being and Society, Nihon Fukushi University, Nagoya, Japan; Charité Universitaetsmedizin Berlin, Germany

## Abstract

**Objectives:**

The purpose of the present study was to examine the potential benefits of additional training in patients admitted to recovery phase rehabilitation ward using the data bank of post-stroke patient registry.

**Subjects and Methods:**

Subjects were 2507 inpatients admitted to recovery phase rehabilitation wards between November 2004 and November 2010. Participants were retrospectively divided into four groups based upon chart review; patients who received no additional rehabilitation, patients who were added with self-initiated off hours training, patients who were added with off hours training by ward staff, patients who received both self-initiated training and training by ward staff. Parameters for assessing outcomes included length of stay, motor/cognitive subscales of functional independent measures (FIM) and motor benefit of FIM calculated by subtracting the score at admission from that at discharge.

**Results:**

Participants were stratified into three groups depending on the motor FIM at admission (≦28, 29∼56, 57≦) for comparison. Regarding outcome variables, significant inter-group differences were observed in all items examined within the subgroup who scored 28 or less and between 29 and 56. Meanwhile no such trends were observed in the group who scored 57 or more compared with those who scored less. In a decision tree created based upon Exhaustive Chi-squared Automatic Interaction Detection method, variables chosen were the motor FIM at admission (the first node) additional training (the second node), the cognitive FIM at admission(the third node).

**Conclusions:**

Overall the results suggest that additional training can compensate for the shortage of regular rehabilitation implemented in recovery phase rehabilitation ward, thus may contribute to improved outcomes assessed by motor FIM at discharge.

## Introduction

Stroke is one of primary debilitating events that affect health status and functional capacity, and is reportedly ranked second or third cause of mortality or condition leading to functional impairments in most developed countries [1]. Japan is no exception that stroke is the first cause of conditions requiring care and is ranked the first in medical expenditure nationwide among the older population [2]. Recent advancement has made various therapeutic options including thrombolytic therapy, intravascular therapy or cerebral protective therapy available for stroke patients, however that does not undermine the significance of rehabilitation for functional recovery. It has been confirmed from previous randomized control trials (RCT) or systematic reviews that providing care in stroke units by multidisciplinary team comprising doctors, nurses, physiotherapist (PT), occupational therapist (OT) and speech therapist (ST) leads to improved clinical outcomes, such as long-term prognosis, activities of daily living at discharge, length of hospital stay [3], [4]. To date, there had been a dearth of multi-center data base for rehabilitation medicine in Japan, which impeded implementation of studies supported by strong evidences. In order to establish rigorous evidences for the quality improvement and to provide rationales for the revision of reimbursement system in stroke rehabilitation, we have been establishing a data bank (DB) of post-stroke patients receiving rehabilitation since 2005, which was supported by a Grant-in-Aid issued from the Ministry of Health, Labor and Welfare for the research project entitled “The development of data bank for stroke rehabilitation”. By November 2011, we collected over 9000 cases from 30 institutions nationwide. In Japan recovery phase rehabilitation ward for patients took effect from the year 2000 and the 2006 revision for reimbursement enabled post stroke patients to receive a maximum of three hours rehabilitation per day by PT, OT and ST. This unique type of ward restricts intake of patient only to medical conditions such as stroke, spinal injuries, head trauma, hip fractures or disuse syndrome. In addition, there are specific regulations regarding the admission criteria, including term of admission. For example, stroke patients have to be admitted within two months after the onset of stroke with maximum length of stay limited until 150 days after the onset. Regarding the time of rehabilitation per day, only those who can tolerate three hours rehabilitation per day are eligible for the entry to rehabilitation programs according to the US Agency for Health Care Policy and Research [5], which is in contrast with the policy applied in Japan. On the other hand, there is a study implemented in a stroke unit that indicated the significance of “off hours” training enhanced by multidisciplinary team for improved activities of daily living (ADL) [1], which suggests a potential benefit of such off hours intervention particularly under the situation where there is a limitation in authorized volume of training. The “OFF hours” training comprises self-initiated training and training by ward staff. However, little attention to date has been paid to off hours intervention and its effect on functional prognosis [6], [7]. As suggested in a recent meta-analysis, the importance of off hours training is a subject to be investigated through further research [8]. Although previous studies regarding off hours training focused on self-initiated training mainly led by patients themselves, there is also a necessity to evaluate additional training provided by ward staff. No studies so far had examined the effect of off hours training (self-initiated training, training by ward staff or both) in recovery phase rehabilitation wards uniquely introduced in Japan.

There is a difficulty in carrying out RCT in rehabilitation medicine. Therefore as an alternative method of investigation, well-designed comparative research with larger samples is considered significant [9]. However, there has been a concern about external validity in previous reports with such method since many of them either came from single institution or had not examined reproducibility in other samples of patients [9], [10]. Thus in the present study, in order to endorse external validity, we obtained observational data from multiple sources and randomly assigned the individual data into two groups and examined whether the equation model formulated in one group can also predict the outcomes in the other with statistical significance.

The purpose of the present study was to examine potential benefit of off-hours rehabilitation involving self-initiated training by patients themselves, and training by ward staff in patients admitted to recovery phase rehabilitation wards using the DB of post-stroke patients registered during the term of observation.

## Subjects and Methods

**Figure 1:**
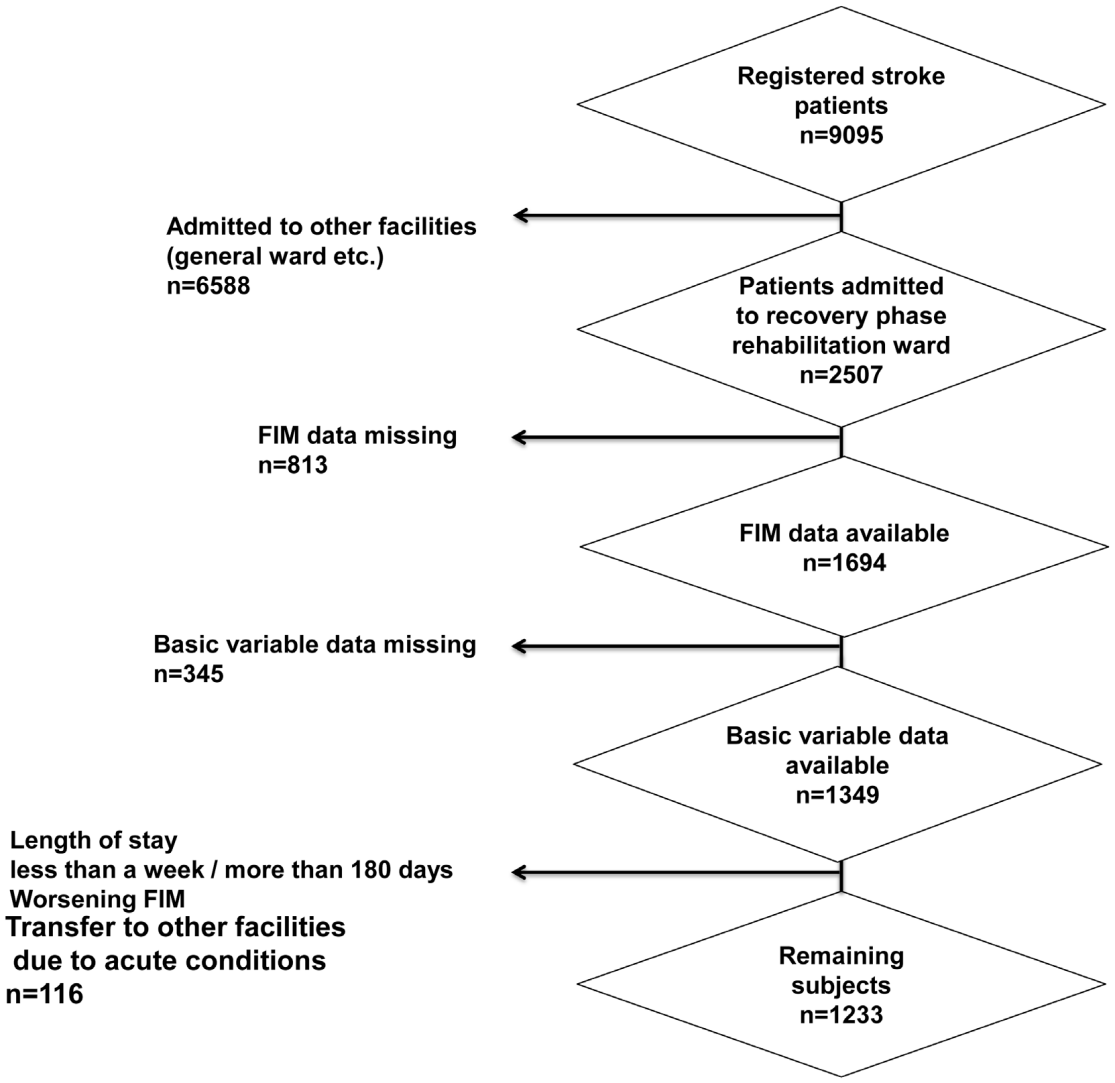
Flow chart showing selection procedure of participants.

The present study was a secondary analysis of the DB of post-stroke patients registered between November 2004 and November 2010. Subjects were 2507 inpatients admitted to recovery phase rehabilitation wards out of 9095 patients registered in post-stroke DB. The DB was managed by the Japan Association of Rehabilitation Database (JARD) and the data was provided after the research protocol was approved by the institutional review board. Thus the data is not publicly available but only those who obtained the approval were authorized access to the DB. Of the subjects, those whose essential data (age, sex, FIM, record of self-initiated off hours training) were either absent or missing in more than 40% of inpatients, length of stay being either less than 7 days or more than 180 days, venue of rehabilitation changed due to acute medical conditions, FIM scores at discharge deteriorated were excluded, eventually 1233 inpatients were subjected to analyses (Fig. 1). Variables included age, sex and type of stroke as basic information. The followings were also included; number of days after admission, number of informal caregivers (none, single person, more than 2 people), total volume of PT and OT counted by Formal Therapy Unit (FTU) and FTU per day were also calculated (1FTU is equivalent to 20 minute Formal Therapy). Parameters for assessing outcomes included length of stay, motor FIM/cognitive FIM and motor benefit of FIM calculated by subtracting the score at admission from that at discharge. Participants were retrospectively divided into four groups based upon chart records; patients who received no additional rehabilitation (no additional training), patients who were added with self-initiated off hours training (self-initiated training), patients who were added with off hours training by ward staff (training by ward staff), patients who received both self-initiated off hours training and training by ward staff (dual additional training) and their outcomes assessed by parameters aforementioned were compared.

### Statistical Analysis

Age, length of stay, number of days after the onset of stroke until discharge, FTU, FTU/day, motor FIM, cognitive FIM and motor benefit of FIM of the four intervention groups were compared using analysis of variance followed by Tukey’s post-hoc test.It is known that improvement of ADLs during admission can be higher in patients whose physical independence at admission is intermediate compared with patients who have either low or high physical independence, thus exhibits reverse U-shaped trend [11]. Therefore, subjects were divided equally into three subgroups based on the motor FIM at admission for group comparison. Categorical data (sex, types of stroke and presence of informal caregivers) of the four groups were compared using chi-square test. In order to clarify contributing factors to motor FIM at discharge after possible confounding factors (presence of informal caregivers, motor FIM at admission and cognitive FIM at admission) having been adjusted, a decision tree analysis was carried out, making measured variables at admission that indicated significant inter-group differences by univariate analysis explanatory variables. In the present study Exhaustive Chi-Squared Automatic Interaction Detection (ECHAID) was adopted for the analysis. ECHAID is a commonly used algorithm of classification tree analysis that employed multi-contingency tables of Chi-squared significant test to identify optimal splits [12]. In order to avoid over fitting, we specified the growing depth of 3 with the parent node having at least 100 subjects and a child node at least 50 subjects. Gains and index charts were constructed to identify the nodes with a relatively high probability. The statistics of misclassification risk was used to assess the prediction results. Primary outcomes to evaluate the effectiveness of additional training was motor FIM at discharge. Motor FIM on admission, the motor FIM at discharge were automatically divided into three ordinal scales (lower tertile; ≦55, mid tertile; 56–79, upper tertile; 80≦) in order for the calculations to fit into the decision tree created. To ensure the validity of the analysis, split-sample validation method was adopted in the present study. In brief, subjects were randomly divided into two groups. Decision trees analysis was carried out in one group and whether the equation obtained in the study group can be applicable in another group (validation group) was examined. All the analyses were carried out using a statistical software package (SPSS version 19.0 for Windows, Chicago IL, USA) and a p value of <0.05 was adopted to show statistical significances. All the personal data were coded deleting any information related to personal identification in order to secure anonymity of the study and the study protocol was approved by the ethical committee of the Japan Society for Rehabilitation Medicine.

## Results

### 1. Group Comparison

Table 1 compares variables of subjects stratified into three groups depending on the motor FIM at admission (≦28, 29∼56, ≧57). Subjects who scored 28 or less showed significant inter-group differences in the type of stroke, age and the interval between the onset and admission. A post-hoc analysis indicated that dual training group was younger and had shorter interval between the onset and admission relative to self-initiated training group and training by ward staff group. Subjects who scored between 29 and 56 showed similar trend in variables examined with those with lower tertile. Meanwhile in subjects who scored 57 or more on the motor FIM at admission, types of stroke and age showed inter-group differences with self-initiated training group and dual additional training group being younger relative to no additional training group.

**Table 1:** Characteristics of participants stratified by motor subscales of FIM at admission.

Motor subscales of FIM at admission ≦28 (n = 427)							
		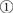 No additional training	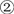 Self-initiated traning	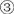 Training by ward staff	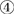 Dual training	p value†	multiple
		(n = 62)	(n = 7)	(n = 203)	(n = 155)		comparison‡
Sex	Male	33(14.2%)	2(0.9%)	113(48.7%)	84(36.2%)	0.59	
	Female	29(14.9%)	5(2.6%)	90(46.2%)	71(36.4%)		
Type fo stroke	CI	38(14.7%)	3(1.2%)	132(51.2%)	85(32.9%)	0.02	
	CH	17(13.0)	2(1.5%)	50(38.2%)	62(47.3%)		
	SAH etc.	7(18.4%)	2(5.3%)	21(55.3%)	8(21.1%)		
Informal care	NIC	17(18.5%)	2(2.2%)	42(45.7%)	31(33.7%)	0.27	
resources	OIC	20(13.7%)	4(2.7%)	64(43.8%)	58(39.7%)		
	MTIC	19(10.6%)	1(0.6%)	94(52.5%)	65(36.3%)		
Age		74.5±9.9	73.0±8.0	75.3±9.2	69.4±11.8	<0.001	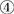 < 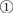 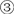
Days after onset at admission		41.1±25.0	48.6±47.3	39.9±21.2	30.9±16.4	<0.001	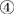 < 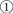 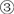
**Motor subscales of FIM at admission 29∼56 (n = 418)**							
		** 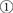 No additional training**	** 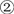 Self-initiated traning**	** 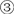 Training by ward staff**	** 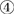 Dual training**	**p value** †	**multiple**
		**(n = 63)**	**(n = 16)**	**(n = 71)**	**(n = 268)**		**comparison** ‡
Sex	Male	50(18.9%)	13(4.9%)	43(16.2%)	159(60.0%)	0.01	
	Female	13(8.5%)	3(2.0%)	28(18.3%)	109(71.2%)		
Type of stroke	CI	27(10.2%)	11(4.1%)	58(21.8%)	170(63.9%)	<0.001	
	CH	31(13.9%)	2(0.9%)	11(8.9%)	79(64.2%)		
	SAH etc.	5(17.2%)	3(10.3%)	2(6.9%)	19(65.5%)		
Informal care	NIC	19(17.3%)	6(5.5%)	23(20.9%)	62(57.3%)	0.46	
resources	OIC	17(12.4%)	4(2.9%)	23(16.8%)	93(67.9%)		
	MTIC	26(15.7%)	6(3.6%)	23(13.9%)	111(66.9%)		
Age		71.8±9.5	68.1±9.7	75.1±10.7	66.6±12.8	<0.001	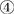 < 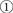 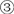
Days after onset at admission		41.9±21.4	34.9±19.4	36.4±18.4	32.0±14.6	<0.001	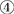 < 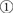 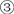
**Motor subscales of FIM at admission 57≦ (n = 388)**							
		** 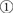 No additional training**	** 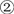 Self-initiated traning**	** 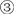 Training by ward staff**	** 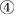 Dual training**	**p value** †	**multiple**
		**(n = 45)**	**(n = 41)**	**(n = 22)**	**(n = 280)**		**comparison** ‡
Sex	Male	23(9.3%)	31(12.6%)	13(5.3%)	180(72.9%)	0.12	
	Female	22(15.6%)	10(7.1%)	9(6.4%)	100(70.9%)		
Type of stroke	CI	28(11.2%)	16(6.4%)	16(6.4%)	190(76.0%)	0.003	
	CH	9(9.8%)	17(18.5%)	2(2.2%)	64(69.6%)		
	SAH etc.	8(17.4%)	8(17.4%)	4(8.7%)	26(56.5)		
Informal care	NIC	12(12.0%)	13(13.0%)	0(0.0%)	75(75.0%)	0.07	
resources	OIC	15(11.7%)	13(10.2%)	9(7.0%)	91(71.1%)		
	MTIC	16(10.6%)	13(8.6%)	12(7.9%)	110(72.8%)		
Age		72.0±11.1	61.7±15.5	70.9±18.2	64.2±13.7	<0.001	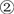 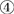 < 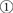
Days after onset at admission		35.4±16.8	34.6±14.4	41.6±26.8	30.4±21.9	0.05	

note:SAH = Subarachnoidal hemorrhage; CI = Cerebral infarction; CH = Cerebral hemorrhage; NIC = No informal caregivers; OIC = One informal caregiver; MTIC = More than two informal caregivers.

† p value for one way analysis of variance.

‡ multiple comparison: digits refer to group numbers (Tukey multiple comparison procedure).

Table 2 shows comparison of variables stratified by the motor FIM at admission. Significant inter-group differences were observed in all items examined within the subgroup who scored 28 or less. A post-hoc analysis revealed that dual training group showed better outcomes compared with training by ward staff group, and dual training group were superior to no additional training group in all parameters apart from the length of stay. Inter-group differences were also observed in a subgroup whose motor FIM at admission were between 29 and 56, and a post-hoc analysis indicated similar results showing that dual training group had better outcomes than training by ward staff group.Meanwhile no such trends were observed by post-hoc analysis in the group who scored 57 or more (upper tertile) compared with those who scored less.

**Table 2:** Outcome parameters of participants at discharge stratified by motor subscales of FIM at admission.

Motor subscales of FIM at admission ≦28 (n = 427)							
	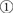 No additionaltraining	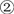 Self-initiatedtraning	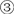 Training by wardstaff	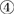 Dual training	p value†		multiple
	(n = 62)	(n = 7)	(n = 203)	(n = 155)			comparison‡
Length of stay	111.3±46.2	105.0±61.4	95.7±43.9	124.9±31.2	<0.001		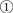 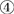 > 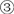
FTU*	358.8±184.0	308.7±252.9	275.3±179.2	474.4±221.4	<0.001		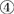 > 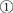 > 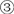
FTU/day*	3.2±1.0	3.2±1.8	2.8±1.2	3.8±1.4	<0.001		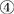 > 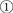 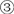
Motor FIM at admission	17.9±5.0	16.9±3.9	16.3±4.2	20.2±4.9	<0.001		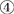 > 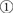 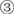
Cognitive FIM at admission	11.4±6.4	15.0±8.6	11.9±6.4	17.9±8.3	<0.001		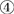 > 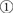 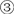
Motor FIM at discharge	31.7±16.3	50.6±22.3	33.0±18.4	52.1±21.4	<0.001		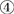 > 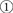 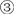
Cognitive FIM at discharge	15.0±7.7	21.7±8.5	15.9±6.4	24.6±8.3	<0.001		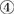 > 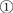 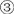
Motor benefit of FIM	13.8±12.8	33.7±22.3	16.7±17.0	31.9±19.7	<0.001		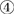 > 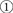 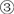 , 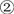 > 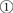
**Motor subscales of FIM at admission 29∼56 (n = 418)**							
	** 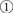 No additional** **training**	** 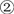 Self-initiated** **traning**	** 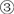 Training by ward** **staff**	** 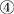 Dual training**	**p value** †		**multiple**
	**(n = 63)**	**(n = 16)**	**(n = 71)**	**(n = 268)**			**comparison** ‡
Length of stay	111.2±47.8	100.4±33.1	88.4±38.1	104.1±37.6	0.01		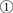 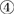 > 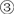
FTU*	398.5±222.4	379.4±171.2	248.3±144.3	405.1±242.7	<0.01		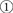 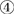 > 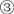
FTU/day*	3.5±1.0	3.8±0.8	2.8±1.1	3.7±1.5	<0.01		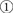 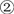 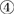 > 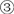
Motor FIM at admission	42.3±8.6	49.5±6.9	39.9±7.5	43.5±8.0	<0.01		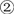 > 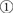 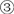 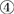 , 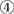 > 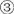
Cognitive FIM at admission	22.1±7.3	24.5±7.5	19.6±5.9	24.7±7.1	<0.01		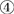 > 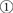 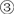 , 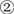 > 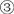
Motor FIM at discharge	61.3±15.7	75.2±7.0	61.0±11.3	72.7±11.5	<0.01		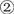 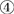 > 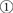 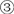
Cognitive FIM at discharge	24.7±7.1	30.0±5.8	22.4±5.8	29.3±5.7	<0.01		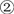 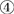 > 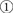 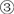
Motor benefit of FIM	19.0±11.6	25.7±8.1	21.1±11.6	29.1±11.3	<0.01		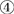 > 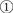 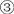
**Motor subscales of FIM at admission 57≦ (n = 388)**							
	** 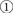 No additional** **training**	** 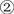 Self-initiated** **traning**	** 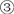 Training by ward** **staff**	** 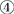 Dual training**	**p value** †	**multiple**	
	**(n = 45)**	**(n = 41)**	**(n = 22)**	**(n = 280)**		**comparison** ‡	
Length of stay	89.3±46.9	69.4±42.4	75.5±33.2	74.6±39.2	0.10		
FTU*	320.7±210.5	251.0±176.3	175.6±78.7	296.0±207.6	0.02	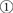 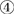 > 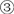	
FTU/day*	3.5±1.1	3.6±1.0	2.4±0.7	3.9±1.6	<0.01	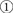 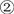 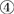 > 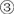	
Motor FIM at admission	71.4±10.3	76.7±9.8	67.3±9.7	70.8±9.2	<0.01	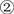 > 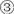 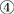	
Cognitive FIM at admission	27.4±6.3	29.0±5.7	19.8±6.3	29.2±5.9	<0.01	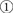 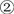 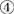 > 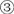	
Motor FIM at discharge	82±7	86.0±6	81.2±7.9	82.2±6.8	0.07	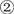 > 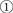 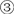 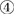	
Cognitive FIM at discharge	29±5.4	31.0±4.2	23.9±5.5	31.6±4.5	<0.01	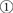 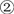 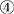 > 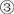 , 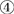 > 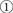	
Motor benefit of FIM	10.6±7.8	9.3±7.9	13.9±8.5	11.4±7.5	0.13		

*FTU: Formal Therapy Unit One unit is equivalent of 20minute rehabilitation.

† p value for one way analysis of variance.

‡ multiple comparison: digits refer to group numbers (Tukey multiple comparison procedure).

### 2. Decision Tree Analysis using ECHAID

**Figure 2:**
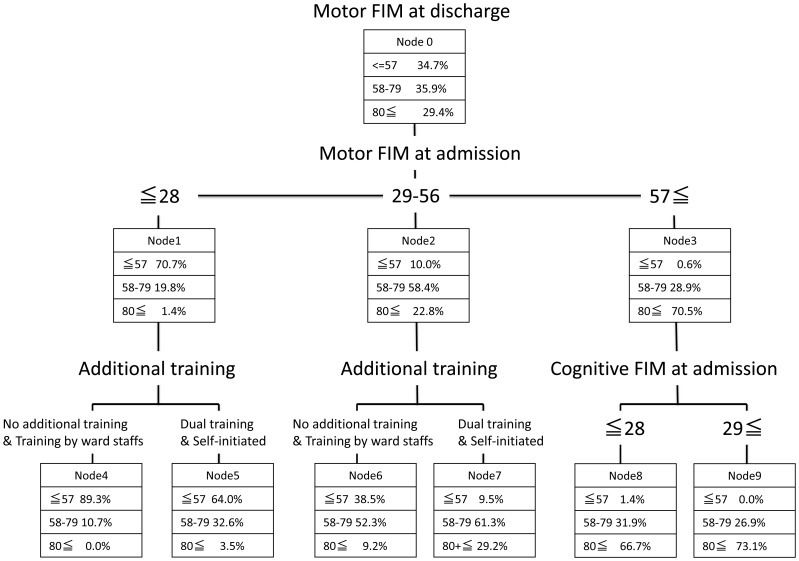
Decision tree for Functional Independence Measure among 1233 stroke patients (Validation Group).

Figure 2 shows a decision tree created based upon ECHAID method. Overall risk estimate for the model in the study group was 0.32, while that in the validation group was 0.31, therefore the analysis was considered appropriate. The variables chosen in the decision tree were the motor FIM at admission,additional trainings,the cognitive FIM at admission. The motor FIM at admission were chosen in the first node, therefore considered most influential on the motor FIM at discharge. For those who scored 56 or less on the motor FIM at admission, no additional training group, training by ward staff and dual training group, self-initiated training group were divided. Meanwhile better cognitive FIM at admission (>28) emerged as a variable to determine improved motor FIM at discharge in those who scored 57 or more on the motor FIM at admission.

## Discussion

The main purpose of the present study was to clarify the effect of additional training other than formal therapy by qualified therapists (PT, OT, ST) on motor FIM at discharge in post-stroke patients. The study utilized multi-center DB of stroke patients and the samples were randomly assigned to either study or validation group. Decision tree analyses were carried out and risk estimates for both groups were compared with an aim to examine whether the model obtained in the study group can be extrapolated in the validation group as well. To date most of studies using decision tree analysis adopt cross validation, which uses random sample out of all subjects for examining validity of the analysis implemented [13], [14]. The limitation about this method is that sampled subjects for validation are included in actual analysis for creating decision tree, and therefore not quite independent. The present analysis was a result from over 1000 cases and was validated by equal number of subjects. Therefore the decision tree created can be considered to exceed in external validity compared with results obtained from conventional methods. The results indicated that in both groups whose motor FIM at admission were either less than 28 or between 29 and 56, those who received both self-initiated training and training by ward staff showed better cognitive and motor FIM at discharge. Furthermore, the decision tree analysis, after adjusting for other possible factors that might affect motor FIM at discharge, also confirmed that implementations of additional training were beneficial in terms of improved outcomes at discharge for those whose motor FIM at admission were below 56. In principle, a factor that appears in the first node has the strongest explanatory power in the decision tree analysis. Thus it was the motor FIM at admission that was most strongly related to the motor FIM at discharge, followed by the implementation of additional training for those in the lower and mid tertile groups of baseline motor FIM. Meanwhile in the upper tertile group, cognitive profiles at admission were more strongly related to the outcomes at discharge than the implementation of additional training. In the upper tertile group, whose overall functional impairment was relatively mild compared with other groups, cognitive capacity affecting attention or concentration to the training assigned may have stronger impact on the efficacy of training than the volume of training. Overall the results suggest that implementation of self-initiated training together with training by ward staff or at least self-initiated training alone might contribute to improved outcomes assessed by motor FIM at discharge, albeit actual contents of off-hours rehabilitation were not available to obtain from the DB. However, given small sample size of patients who implemented self-initiated training, the present results must be interpreted with caution. A previous study by Galvin et al [15].employing a randomized controlled trial (RCT) on the effect of self-initiated training confirmed the improvement of ADLs or easing stress experienced by family. Another report also stressed the efficacy of self-initiated training assisted by patients’ family for improved physical functions of lower extremities and ADLs [7]. A systematic review by Meheroz et al [6]. have demonstrated that off hours repeated use of upper extremities may contribute to improved functions. Previous studies including RCT [15]–[17] that investigated the effect of self-initiated training targeted particular conditions such as first onset, no episode of dementia or restricted severity of hemiparesis, therefore the findings can be applied to conditions meeting inclusion criterion. Meanwhile the results obtained in the present study can be applied to patients with broad conditions of stroke. Regarding the effect of training by ward staff, Indredavik et al [18] stated that one of the advantages of stroke unit compared with general ward is the preventive approaches of secondary complications or disuse syndromes by staff nurses. Studies of stroke unit have shown that multidisciplinary interventions might lead to beneficial outcomes. Likewise, the present study suggested the importance of close collaborations of multidisciplinary staffs by having demonstrated that the implementation of either both self-initiated training and additional training by ward staff or self-initiated training alone was found to be beneficial for improving motor outcomes in post stroke patients admitted to recovery phase rehabilitation ward although the findings cannot directly be applied to any ward accommodating post stroke patients. Possibly due to insufficient availability of physiotherapist, average total time of regular rehabilitation per day in the present study was approximately 70 minutes, which figures fall far short of upper limit of 90 minutes. Additional training can compensate for the shortage, which therefore must have worked effectively for the improvement of motor function as suggested in previous reports [1], [19].

Even though every possible confounding factors had been considered in the present analyses, so-called reverse causality of having chosen subjects who were expected to improve cannot completely be eliminated, which serves as a limitation of the present study. Further analyses adopting propensity score, instrumental variables or RCT would be necessary to control reverse causality. Due to restriction of data availability, actual amount of time and intensity of self-initiated training and training by ward staff were not considered. Comparison of efficacy betweenthe two off hours training groups favored self-initiated training. Elevated motivation of patients themselves, leading to proactive participation to the training, may explain the observed difference although such possible reason remains a speculation under the absence of detailed information about off hours training. Despite external validity of this multi-center study having been warranted, given that many institutions participated in the DB registration had specialists of rehabilitation medicine, and had elevated motivation represented by relatively higher implementation rate of training by ward staff, the present results need to be interpreted with caution. Nonetheless, we believe that the present multi-center study using stroke DB suggested the significance of additional (self-initiated, by ward staff or both) training at least for patients whose ADLs at admission are classified as more than moderately impaired.
